# Supplementary material for: Similarities between action potentials and acoustic pulses in a van der Waals fluid
Source: Sci Rep. 2019 Feb 21;9:2467. doi: 10.1038/s41598-019-38826-x (PMC6385226; doi:10.1038/s41598-019-38826-x)
Supplement: Supplementary file 1 — Supplemental materials [file 41598_2019_38826_MOESM1_ESM.pdf]

Similarities between action potentials and acoustic pulses in a van der Waals fluid

Matan Mussel*†‡ Matthias F. Schneider*

May 22, 2018

Supplemental materials

The supplemental materials are intended to provide interested readers with further mathematical details (section A), additional solutions (section B) and caption to the supplemental movie (section C).

A Model Equations

Model variables and parameters. The fluid considered in this letter is described by six dynamic variables of space and time, that are coupled to one another by three conservation laws (Eq. (1)), two constitutive equations (Eq. (4)), and one inverse relation, $\rho = w^{-1}$. The six variables are listed in Table S1. In addition, the model depends on seven constant parameters, all are measurable physical quantities (Table S2). The van der Waals parameters (m, a, b) are related to the critical point according to

$$w_c = \frac{3b}{m}, \qquad p_c = \frac{a}{27b^2}, \qquad \theta_c = \frac{8a}{27bk_B}. \tag{S1}$$

The scaling of the spatial coordinate h (in the Lagrange frame) was defined according to the critical density ($\bar{w} = w_c$). Finally, $k_B \cong 1.38 \cdot 10^{-23}$ J/K is the Boltzmann constant.

Dimensionless model equations. The critical point and the fluid viscosity parameters were used (Eq. (5)) to introduce the following reduced variables and parameters:

$$\begin{gathered} X \equiv \frac{h}{L}, \qquad \tilde{t} \equiv \frac{t}{T} \\ \tilde{w} \equiv \frac{w}{w_c}, \qquad \tilde{v} \equiv \frac{v}{U}, \qquad \tilde{E} \equiv \frac{E}{U^2}, \\ \tilde{\theta} \equiv \frac{\theta}{\theta_c}, \qquad \tilde{p} \equiv \frac{p}{p_c}, \qquad \tilde{\tau} \equiv \frac{\tau}{p_c}. \end{gathered} \tag{S2}$$

The spatial dimension in the Lagrange frame was normalized by the choice $\bar{w} = w_c$. The dimensionless model equations are

$$\begin{aligned} \partial_{\tilde{t}} \tilde{w} &= \partial_X \tilde{v}, \\ \partial_{\tilde{t}} \tilde{v} &= \partial_X \left(\tilde{\tau}_1 + \tilde{\tau}_2\right), \\ \partial_{\tilde{t}} \tilde{E} &= \partial_X \left(\tilde{\tau}_1 \tilde{v}\right) + \tilde{k} \partial_X^2 \tilde{\theta}, \end{aligned} \tag{S3}$$

with

$$\begin{aligned} \tilde{\tau}_1 &= -\tilde{p} + \partial_X \tilde{v}, \\ \tilde{\tau}_2 &= -\tilde{C} \partial_X^2 \tilde{w}, \end{aligned} \tag{S4}$$

and

$$\begin{aligned} \tilde{p} &= \frac{8\tilde{\theta}}{3\tilde{w} - 1} - \frac{3}{\tilde{w}^2}, \\ \tilde{E} &= \frac{\tilde{v}^2}{2} + \tilde{c}_v \tilde{\theta} - \frac{3}{\tilde{w}} + \frac{\tilde{C}}{2} (\partial_X \tilde{w})^2, \end{aligned} \tag{S5}$$

The model equations (S3)–(S5) depend on three dimensionless parameters: heat capacity, thermal conductivity and capillarity coefficient

$$\tilde{c}_v = \frac{c_v \theta_c}{p_c w_c}, \qquad \tilde{k} = \frac{k \theta_c}{p_c w_c \zeta}, \qquad \tilde{C} = \frac{C}{\zeta^2}. \tag{S6}$$

Note that a physically meaningful solution requires $1/3 < \tilde{w}$ and $0 < \tilde{\theta}$.

Model equations in the Euler frame. The model equations in the Lagrange frame are given in Eq. (1). These equations were transformed into the Euler frame using Eq. (2); i.e., $\partial x / \partial h = w / \bar{w}$, and by replacing the time derivative with the material derivative $\partial_t \to D_t = \partial_t + v \partial_x$

$$\begin{aligned} D_t w &= w \partial_x v, \\ D_t v &= w \partial_x (\tau_1 + \tau_2), \\ D_t E &= w \left[\partial_x (\tau_1 v) + \frac{k}{\bar{w}^2} w \partial_x (w \partial_x \theta) \right], \end{aligned} \tag{S7}$$

and

$$\begin{aligned} \tau_1 &= -p + \zeta \frac{w}{\bar{w}} \partial_x v, \\ \tau_2 &= -C \frac{w}{\bar{w}^2} \partial_x (w \partial_x w). \end{aligned} \tag{S8}$$

In comparison, the classical Navier-Stokes equations in the Euler frame are

$$\begin{aligned} D_t w &= w \partial_x v, \\ D_t v &= w \partial_x \tau_{NS}, \\ D_t E &= w [\partial_x (\tau_{NS} v) + k \partial_x^2 \theta], \end{aligned} \tag{S9}$$

with

$$\tau_{NS} = -p + \zeta \partial_x v. \tag{S10}$$

*Department of Physics, Technical University of Dortmund, 44227 Dortmund, Germany
†Department of Physics, University of Augsburg, 86159 Augsburg, Germany
‡Correspondence to: matan.mussel@physik.uni-augsburg.de

Variable	Name	Units for a 3d material (2d material) in MKS system
w	specific volume	$\frac{m^3}{kg}$ $\left(\frac{m^2}{kg}\right)$
v	velocity	$\frac{m}{s}$
E	specific total energy	$\frac{m^2}{s^2}$
p	pressure	$\frac{N}{m^2}$ $\left(\frac{N}{m}\right)$
θ	temperature	K
ρ	density	$\frac{kg}{m^3}$ $\left(\frac{kg}{m^2}\right)$

Table S1: Model variables

Parameter	Name	Units for a 3d material (2d material) in MKS system
m	mass of a fluid particle	kg
a	average attraction between fluid particles	Nm^4 $\left(Nm^3\right)$
b	volume exclusion by the particles	m^3 $\left(m^2\right)$
ζ	dilatational viscosity	$\frac{kg}{m \cdot s}$ $\left(\frac{kg}{s}\right)$
c_v	specific heat capacity at constant volume	$\frac{J}{kg \cdot K}$
k	thermal conductivity	$\frac{J}{m \cdot s \cdot K}$ $\left(\frac{J}{s \cdot K}\right)$
C	capillarity coefficient	$\frac{kg^2}{m^2 \cdot s^2}$ $\left(\frac{kg^2}{s^2}\right)$

Table S2: Model constant parameters

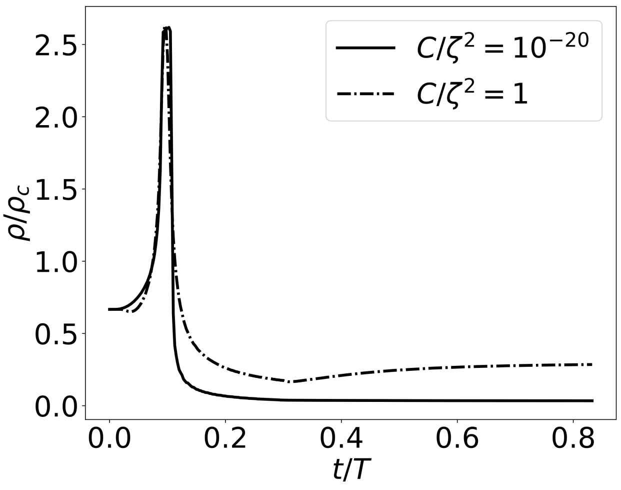

Figure S1: Comparison of pulse shape at two different values of the capillarity coefficient, $C/\zeta^2 = 10^{-20}$ (solid line) and 1 (dotted-dashed line). Excitation amplitude was $\tilde{p}_{exc} = 120$ and 300, respectively. Duration of excitation was $\tilde{t}_0 = 0.3$ for both curves. Pulse was measured at x/L=0.5 and 0.8, respectively. Other parameters appear in caption of Fig. 2.

B Pulse solution in a vdW fluid model

Capillarity coefficient and the line tension. Gibbs approached the phase boundary as an infinitesimal line that separates the two phases. The energy stored in a phase boundary of length L and line tension γ is $E = \int \gamma(l)dl = \gamma L$. In contrast, van der Waals approached the phase transition as a continuous change, and the phase boundary, therefore, has a finite width. Van der Waals suggested an energy term that depends on the density gradient

$$E = \int K \left(\frac{\partial \rho}{\partial x}\right)^2 d^2x. \tag{S11}$$

For a phase boundary of width ℓ and a difference in density $\Delta\rho$ between the two phases, the energy stored in the phase boundary is $E \sim K\frac{\Delta\rho^2}{\ell^2}\ell L$. Therefore, the capillarity coefficient is related to the line tension according to

$$K \sim \frac{\gamma \ell}{\Delta\rho^2}. \tag{S12}$$

Using Slemrod‘s ansatz, the capillarity coefficient is $C \sim \frac{\gamma \ell \rho_c^3}{\Delta\rho^2}$ from scaling arguments. Assuming $\ell \sim 10^{-9} - 10^{-6}$ m, $\Delta\rho \sim \rho_c \sim 10^{-6}\ kg/m^2$ and $\gamma \sim 10^{-12}$ N (Benvegnu and McConnell, J Phys Chem, 1992) we obtain $C \sim 10^{-27} - 10^{-24}\ kg^2/s^2$. However, in the numerical calculation we have used a larger value ($C/\zeta^2 \sim 1$), to avoid sharp gradients in density. This did not have qualitative effect on the properties of sound pulses near phase transition as evident in Fig. S1.

Effect of stimulation parameters on the pulse characteristics. Figure S2a shows the effect of stimulation duration on the shape of the density pulse, as measured at distance x/L=1. It was evident that the compression part of the density pulse was not affected much and maintained a pulse duration of ≈0.1T. On the other hand, the subsequent rarefaction monotonically decreased its density with stimulation duration. The density, pressure and temperature field solutions as a function of space (x-axis) and time (y-axis) are shown in Figs S2b,c,d, respectively, for stimulation duration of t_0/T=0.4. It was evident that the pulse maintained a recognizable shape for distances larger than 10L. An increase of the stimulation width (λ) resulted in similar observations (Fig. S2e).

C Caption of supplemental movie

<u>Movie S1:</u> Collision between two propagating pulses is demonstrated in the density, pressure and temperature fields. At the peak of the collision a short burst of pressure is obtained (t/T=0.2). The pulses annihilate afterwards. Computational details are given in caption of Fig. 5.

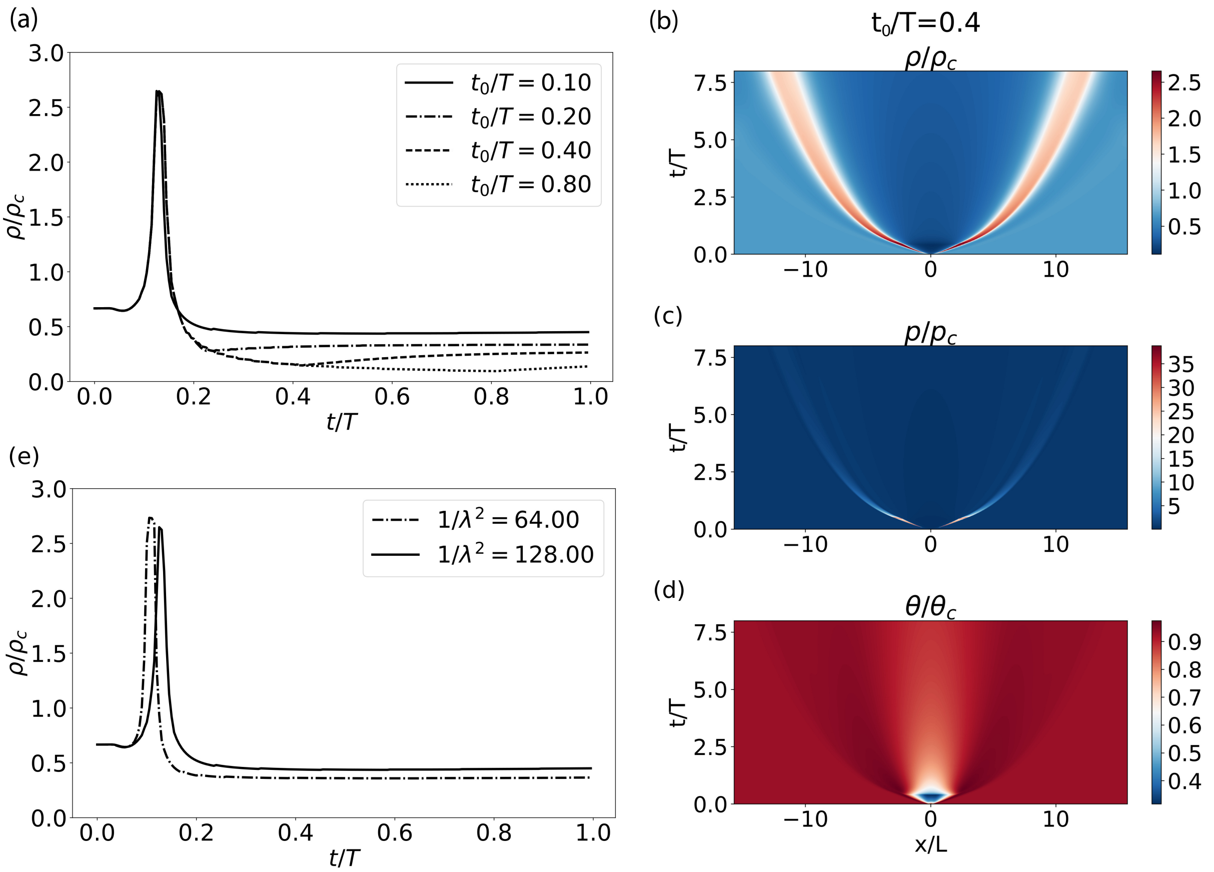

Figure S2: (a) Variation of stimulation duration (t_0) did not affect the duration of the compression part of the density pulse, as measured at distance x/L=1 from the excitation point. The subsequent rarefaction, however, was altered. (b) Density, (c) pressure and (d) temperature solutions as a function of space (x-axis) and time (y-axis) upon stimulation with t_0/T=0.4. Pulse maintains a recognizable shape for distances larger than 10L. (e) The shape of the pulse did not change much with an increase in stimulation width. Initial density was $\tilde{\rho}_0 = 0.67$, and numerical calculation was conducted with 2048 grid points, x-domain $[-5\pi, 5\pi]$ and dt=10^{-3}. Other parameters appear in the caption of Fig. 2.
